# Maternal mortality in the context of COVID-19: an ecological study comparing maternal mortality ratios before and during the pandemic, Minas Gerais, Brazil, 2010-2021

**DOI:** 10.1590/S2237-96222026v35e20251008.en

**Published:** 2026-05-08

**Authors:** Lívia Maria Gomes Lopes, Juliana Bottoni de Souza, Deborah Carvalho Malta, Flávia Bulegon Pilecco

**Affiliations:** 1 Universidade Federal de Minas Gerais Programa de Pós-Graduação em Saúde Pública Belo Horizonte MG Brazil Universidade Federal de Minas Gerais, Programa de Pós-Graduação em Saúde Pública, Belo Horizonte, MG, Brazil; 2 Universidade Federal de Minas Gerais Escola de Enfermagem Belo Horizonte MG Brazil Universidade Federal de Minas Gerais, Escola de Enfermagem, Belo Horizonte, MG, Brazil; 3 Universidade Federal de Minas Gerais Departamento de Enfermagem Materno Infantil e Saúde Pública Belo Horizonte MG Brazil Universidade Federal de Minas Gerais, Departamento de Enfermagem Materno Infantil e Saúde Pública, Belo Horizonte, MG, Brazil; 4 Universidade Federal de Minas Gerais Departamento de Medicina Preventiva e Social Belo Horizonte MG Brazil Universidade Federal de Minas Gerais, Departamento de Medicina Preventiva e Social, Belo Horizonte, MG, Brasil

**Keywords:** Maternal Mortality, COVID-19, Pandemics, Health Status Disparities, Ecological Studies, Mortalidad Materna, COVID-19, Pandemias, Disparidades en el Estado de Salud, Estudios Ecológicos

## Abstract

**Objective:**

To investigate changes in maternal mortality during the COVID-19 pandemic according to sociodemographic, regional and obstetric care characteristics in Minas Gerais state, and the correlation of the Maternal Mortality Ratio (MMR) with socioeconomic indicators of macro-region of residence.

**Methods:**

The study compared Mortality Information System data between 2010-2019 and 2020-2021. The MMR and its percentage change were calculated according to age, schooling, race/skin color, number of prenatal consultations/trimester of prenatal care initiation and health macro-region. We assessed the correlation between the MMR and macro-region socioeconomic variables (Gross Domestic Product [GDP] per capita, Brazilian Deprivation Index and Schooling).

**Results:**

1,488 deaths were recorded. The MMR increased by 62.5% during the pandemic, especially among women aged 20 to 34 (64.5%), White women (77.7%), women with lower education levels (134.9%), fewer prenatal visits (102.2%), those who started prenatal care in the 1st trimester (58.1%), and those living in the Northwest macro-region (241%). However, the highest MMRs were found in women aged 35 or older (125.1/100,000 live births), Black women (90.5/100,000 live births), women with lower education levels (151.5/100,000 live births), women with fewer prenatal visits (181.2/100,000 live births), women who started prenatal care in the 1st trimester (61.8/100,000 live births), and women living in the Northeast macro-region (122.3/100,000 live births). We found no correlation between socioeconomic indicators and the MMR during the pandemic.

**Conclusion:**

The percentage increase in the ratio during the pandemic was greater in groups and regions with historically lower MMRs. Timely recognition of pregnant/postpartum women as a risk group in health crises can reduce preventable maternal deaths.

## Resumen

**Objetivo:**

Investigar los cambios en la mortalidad materna durante la pandemia de COVID-19 según las características sociodemográficas, regionales y de atención obstétrica en el estado de Minas Gerais, y la correlación de la Razón de Mortalidad Materna (RMM) con los indicadores socioeconómicos de la macrorregión de residencia.

**Métodos:**

El estudio comparó datos del Sistema de Información de Mortalidad entre 2010-2019 y 2020-2021. Se calcularon la RMM y la variación porcentual según edad, educación, raza/color de piel, número de consultas/trimestre de inicio de la atención prenatal y macrorregión de salud. Se evaluó la correlación entre la RMM y las variables socioeconómicas de la macrorregión (Producto Interno Bruto [PIB] per cápita, Índice de Privación Brasileño y Educación).

**Resultados:**

Se registraron 1488 muertes. La tasa de mortalidad materna aumentó 62,5% durante la pandemia, especialmente entre las mujeres de 20 a 34 años (64,5%), las blancas (77,7%), las de menor nivel educativo (134,9%), las que acudieron menos a consultas prenatales (102,2%), las que iniciaron la atención prenatal en el primer trimestre (58,1%) y las residentes de la macrorregión Noroeste (241%). Sin embargo, las RMM más altas se observaron en mujeres de 35 años o más (125,1/100.000 nacidos vivos), mujeres negras (90,5/100.000 nacidos vivos), mujeres con menor nivel educativo (151,5/100.000 nacidos vivos), mujeres con menos visitas prenatales (181,2/100.000 nacidos vivos), mujeres que iniciaron la atención prenatal en el primer trimestre (61,8/100.000 nacidos vivos) y mujeres residentes en la macrorregión Noreste (122,3/100.000 nacidos vivos). No se encontró correlación entre los indicadores socioeconómicos y la RMM durante la pandemia.

**Conclusión:**

El aumento porcentual de la razón durante la pandemia fue mayor en los grupos y regiones con RMM históricamente más bajos. El reconocimiento oportuno de las mujeres embarazadas/puérperas como grupo de riesgo en crisis sanitarias puede reducir las muertes maternas prevenibles.

**Ethical aspects**

This research respected ethical principles, having obtained the following approval data:

Research ethics committee: Universidade Federal de Minas Gerais

Opinion number: 5,910,012

Approval date: 24/2/2023

Certificate of submission for ethical appraisal: 66320122.1.0000.5149

Informed consent form: Waived.

## Introduction

Maternal mortality is a recurring public health problem, especially in low- and middle-income countries. In Latin America, nine out of ten such deaths are preventable if proven effective measures and recommendations are applied, such as quality maternal care (including in obstetric emergencies), universal access to contraceptive methods and addressing inequalities in access to health [1].

The COVID-19 pandemic disproportionately affected pregnant and postpartum women, increasing the Maternal Mortality Ratio (MMR), especially in developing countries, including Brazil [2-5], where the MMR increased from 57.9 deaths per 100,000 live births in 2019 to 74 in 2020 [6] and to 117.4 deaths per 100,000 live births in 2021 [7]. This increase in the MMR distances Brazil from achieving its Sustainable Development Goals target of no more than 30 deaths per 100,000 live births by 2030, set by Brazil and agreed with the United Nations [8].

In addition to maternal deaths directly caused by COVID-19, the economic crisis and restrictions on public investment in health are considered to have intensified pre-existing problems in the Brazilian health system [9], exacerbating the indirect effects of the pandemic. Access to various health services was impacted by the overload and diversion of health professionals, beds and financial resources to combat the disease [10]. Regarding obstetric care, there was an aggravation of chronic problems, such as insufficient resources, limitations and inequalities in access to qualified prenatal care and intensive care [11]. Furthermore, the omissions and negligence of the Federal government in managing the pandemic, dissemination of fake news and demonstrably ineffective medications, as well as the delay in acquiring vaccines to control the epidemic [2] contributed to the worsening of the crisis in Brazil.

Differential risks in maternal mortality related to socioeconomic [4,12] and demographic [3,13] factors were identified in national contexts during the pandemic. To date, no studies have been found that explore temporal variations in the MMR related to the COVID-19 pandemic in a Brazilian state as heterogeneous as Minas Gerais, which has not achieved significant reductions in the MMR in recent years [6]. However, Minas Gerais has reliable data on maternal deaths, with investigation rates of deaths of women of childbearing age above 95% up to 2019 [7]. This form of investigation enables the recovery of maternal deaths not originally reported, this being essential for an MMR estimate closer to reality.

The objective of this study was to investigate changes in maternal mortality during the COVID-19 pandemic according to sociodemographic, regional and obstetric care characteristics in Minas Gerais, and the correlation of maternal mortality with socioeconomic indicators of macro-region of residence. 

## Methods

### Design

This was an ecological study comparing maternal mortality rates between the COVID-19 pre-pandemic and pandemic periods in Minas Gerais, between 2010 and 2021.

### Setting

Maternal death is considered to be that which occurs during or up to 42 days after the end of pregnancy, the underlying cause of which is coded in Chapter XV of the 10^th^ revision of the International Classification of Diseases (ICD-10) [14]. Codes O00 to O99 were considered, except O96 and O97, which refer to late maternal death and death due to sequelae of direct obstetric causes. Codes A34, B20-B24, F53, D39.2, E23.0 and M83.0 were also included, provided that the deaths were related to the pregnancy-puerperal state and occurred up to 42 days after delivery [14].

Two periods were compared: pre-pandemic (2010-2019) and pandemic (2020-2021). The adoption of a ten-year time series in the period prior to the pandemic aimed to ensure greater statistical stability, resulting from an adequate number of deaths.

### Participants 

All maternal deaths recorded on the Mortality Information System, of women residing in Minas Gerais, that occurred between 2010 and 2021. As this is a population-based census study, no sample size calculation was performed. From 2010 to 2021, 1,488 maternal deaths were recorded.

### Variables

The MMR (number of maternal deaths/number of live births in the same period x 100,000) was calculated for both the period before the COVID-19 pandemic (2010-2019) and the pandemic period itself (2020-2021), and the percentage change in the MMR was calculated as: [(final MMR - initial MMR) / initial MMR] x 100. The MMR and its percentage change were calculated for the following variables:

- age (in years: ≤19; 20-34; ≥35);
- schooling (in years of study: 0-7; ≥8);
- race/skin color (White; Black; mixed-race);
- number of prenatal consultations (0-6; ≥7);
- term in which the first prenatal consultation took place (1^st^ term; 2^nd^-3^rd^ term); and
- Health macro-region according to the Minas Gerais Regionalization Master Plan (Central; South Central; Jequitinhonha; East; East Southern; Northeast; Northwest; North; West; Southeast; South; Triângulo do Norte; Triângulo do Sul; Vale do Aço) [15].

With regard to the race/skin color variable, the Asian (n=3) and Indigenous (n=6) categories were not presented in the sociodemographic variables results table due to the small number of observations in the period. Regarding the prenatal care start trimester variable, the year 2010 was not considered due to inconsistencies in this field on the Live Birth Information System.

### Data source and measurement 

The Mortality Information System data, including the maternal death investigation module variables, were obtained by means of a formal request made to the Minas Gerais State Health Department. The data were made available in March 2023. The number of live births, used to calculate the MMR, was accessed via the Live Births Information System, available from the Brazilian Unified Health System Department of Information Technology Health Information System [7].

### Statistical analysis 

Heatmap graphs were built to present the MMRs and the percentage changes in MMRs by health macro-region, whereby the color varies in shades of red (Figure 1). As the shade of red intensifies, the MMRs are higher or the percentage increases are greater. Conversely, less intense shades of red correspond to lower MMRs and greater reductions or smaller percentage increases.

**Figure 1:**
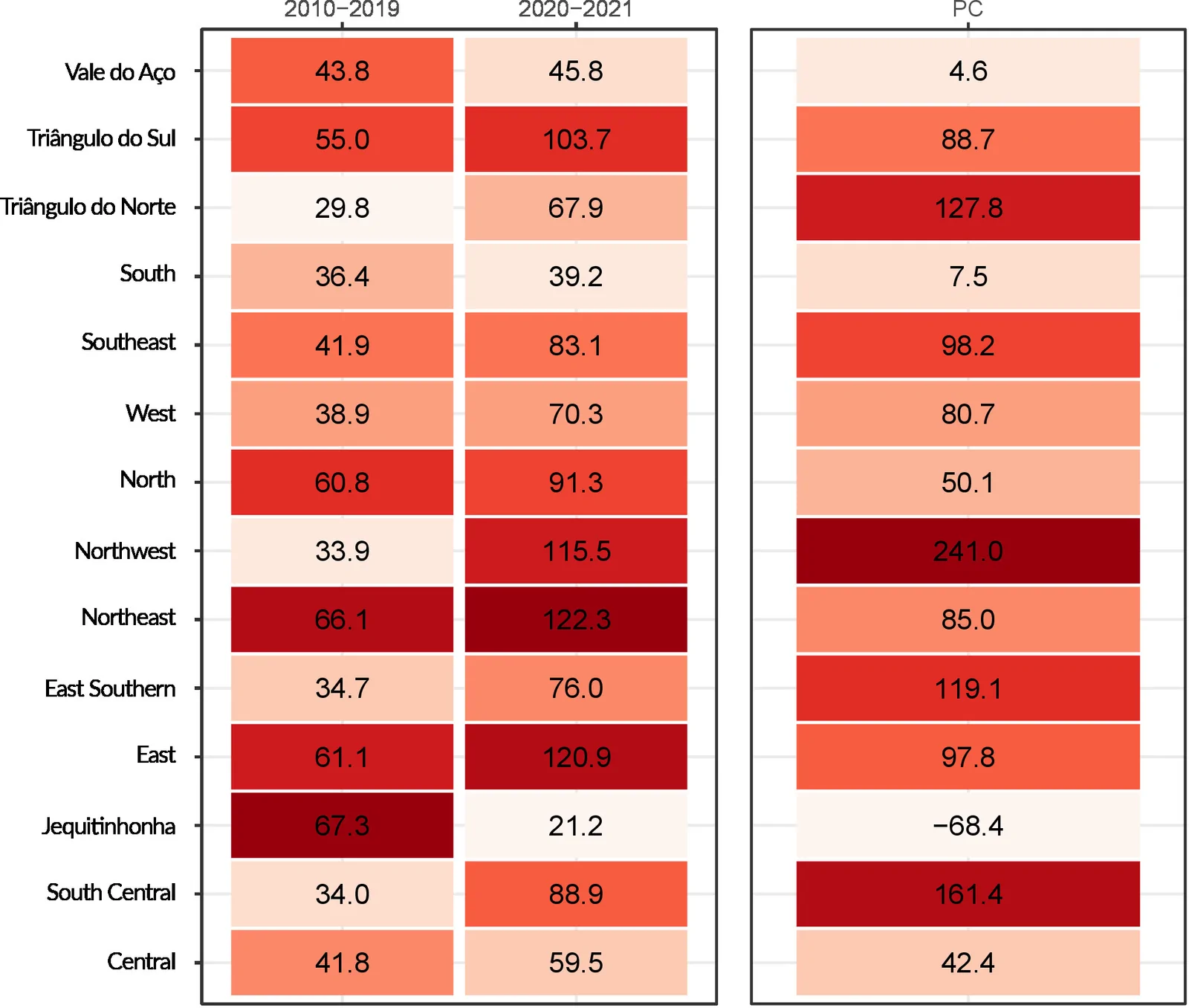
Maternal mortality ratios in the health macro-regions before and during the COVID-19 pandemic and percentage change (PC) between the periods. Minas Gerais, 2010-2021 (n=1,488)

In order to describe socioeconomic disparities between regions, we used Gross Domestic Product (GDP) per capita [16], the Brazilian Deprivation Index [17] and the percentage of young people (18-24 years old) who did not complete elementary education (low schooling). The average GDP per capita values ​​(2017-2019) were obtained from the database of the Brazilian Institute of Geography and Statistics (IBGE) [18]; the median Brazilian Deprivation Index was obtained from the Center for Health Data and Knowledge Integration [17]; while the schooling percentage was obtained from the Brazilian Unified Health System Department of Information Technology Health Information System (data referring to the 2010 IBGE census) [7]. A graphical analysis of the correlation between these indicators and the MMR by health macro-region was performed for the periods analyzed, additionally calculating the Pearson correlation coefficient (R) [19], adopting a 5% significance level. The analyses were conducted using Stata software, version 14, and R software with the aid of the Rgeoda package.

Finally, the percentage of investigations and the proportion of deaths of women of childbearing age recategorized as post-investigation maternal deaths were described, based on a comparison of the original underlying cause and the final underlying cause. The proportional distribution of deaths according to the type of cause (direct, indirect and undetermined) and by ICD-10 groups for deaths in pregnancy, childbirth and the puerperium (ICD-MM) [14] (Supplementary Table 1) were described. The ICD-MM defines, in a subgroup, causes of death that should not be used as the underlying cause. In this study, they were reallocated to group 5 – other obstetric complications. Maternal deaths due to COVID-19 are identified by the following underlying causes: O98.5 + U07.1; O98.5 + U07.2; O98.5 + U09.9; O99.8 + U10.9; O98.5 + U92.1 [6].

**Table 1:** Specific Maternal Mortality Ratio (MMR) (per 100,000 live births) according to selected variables before and during the COVID-19 pandemic and percentage change between the periods. Minas Gerais, 2010-2021 (n=1,488)

Sociodemographic and health care variables	2010-2019 MMR (n)	2020-2021 MMR (n)	Change between periods %
Total	43.8 (1,140)	71.1 (348)	62.5
**Age group **(years)			
≤19	25.9 (102)	34.6 (19)	33.6
20-34	38 (695)	62.5 (214)	64.5
≥35	89.7 (343)	125.1 (115)	39.5
**Schooling **(years)^a^			
0-7	64.5 (342)	151.5 (77)	134.9
≥8	27.9 (563)	53.6 (234)	92.1
**Race/skin color^b^**			
White	40.4 (365)	71.8 (113)	77.7
Black	88.5 (162)	90.5 (43)	2.3
Mixed-race	41.4 (553)	68.6 (185)	65.7
**Prenatal consultations^c^**			
0-6	89.6 (596)	181.2 (177)	102.2
≥7	24.4 (470)	40.4 (158)	65.6
**Term prenatal care started^d^**			
1^st^	39.1 (689)	61.8 (254)	58.1
2^nd^-3^rd^	43.1 (146)	56.9 (34)	32.0

^a^ Blank/unknown fields accounted for 20.6% (2010-2019) and 10.6% (2020-2021); ^b^ Blank/unknown fields accounted for 4.7% (2010-2019) and 1.2% (2020-2021); ^c^ Blank/unknown fields accounted for 6.5% (2010-2019) and 3.7% (2020-2021); ^d^ Blank/unknown fields accounted for 18.1% (2010-2019) and 17.2% (2020-2021).

## Results

We assessed 1,488 death certificates covering the period 2010 to 2021. In Minas Gerais, there was a significant increase in the MMR during the pandemic (62.5%), rising from 43.8 (2010-2019) to 71.1 deaths per 100,000 live births (2020-2021). The largest increases occurred among women aged 20 to 34 (64.5%), with lower education (up to 7 years of schooling) (134.9%), of White (77.7%) and brown (mixed-race) (65.7%) race/skin color, with fewer prenatal visits (0-6 visits) (102.2%), and with prenatal care initiated in the 1^st^ trimester (58.1%). The highest MMRs during the pandemic, however, were in women aged 35 or older (125.1 per 100,000 live births), with less than 8 years of schooling (151.5 per 100,000 live births), of Black race/skin color (90.5 per 100,000 live births), who had 0 to 6 prenatal visits (181.2 per 100,000 live births) and who started prenatal care in the 1^st^ trimester (61.8 per 100,000 live births) (Table 1).

The MMR increased during the pandemic period in most macro-regions. The exception is the Jequitinhonha macro-region, the only one that showed a reduction. During the pandemic period, the largest increases were in the Northwest (241.0%) and South Central (161.4%) macro-regions, and the smallest in the Vale do Aço (4.6%) and Southern (7.5%) macro-regions. The highest MMRs were in the Northeast (122.3 per 100,000 live births) and East (120.9 per 100,000 live births) macro-regions (Figure 1).

Correlation analysis showed that, in the period 2010-2019, the MMR increased as the median health macro-region Brazilian Deprivation Index increased (R 0.73; p-value 0.003) and with the higher percentage of young people who did not complete elementary education (R 0.69; p-value 0.006). There was no statistically significant correlation with GDP per capita (R -0.44; p-value 0.110). During the pandemic period, there were no statistically significant correlations between MMRs and the socioeconomic indicators assessed (Figure 2). Even excluding Jequitinhonha from the analysis, due to its very different results from those expected during the pandemic period, statistically significant correlations continued to be absent during this period.

**Figure 2:**
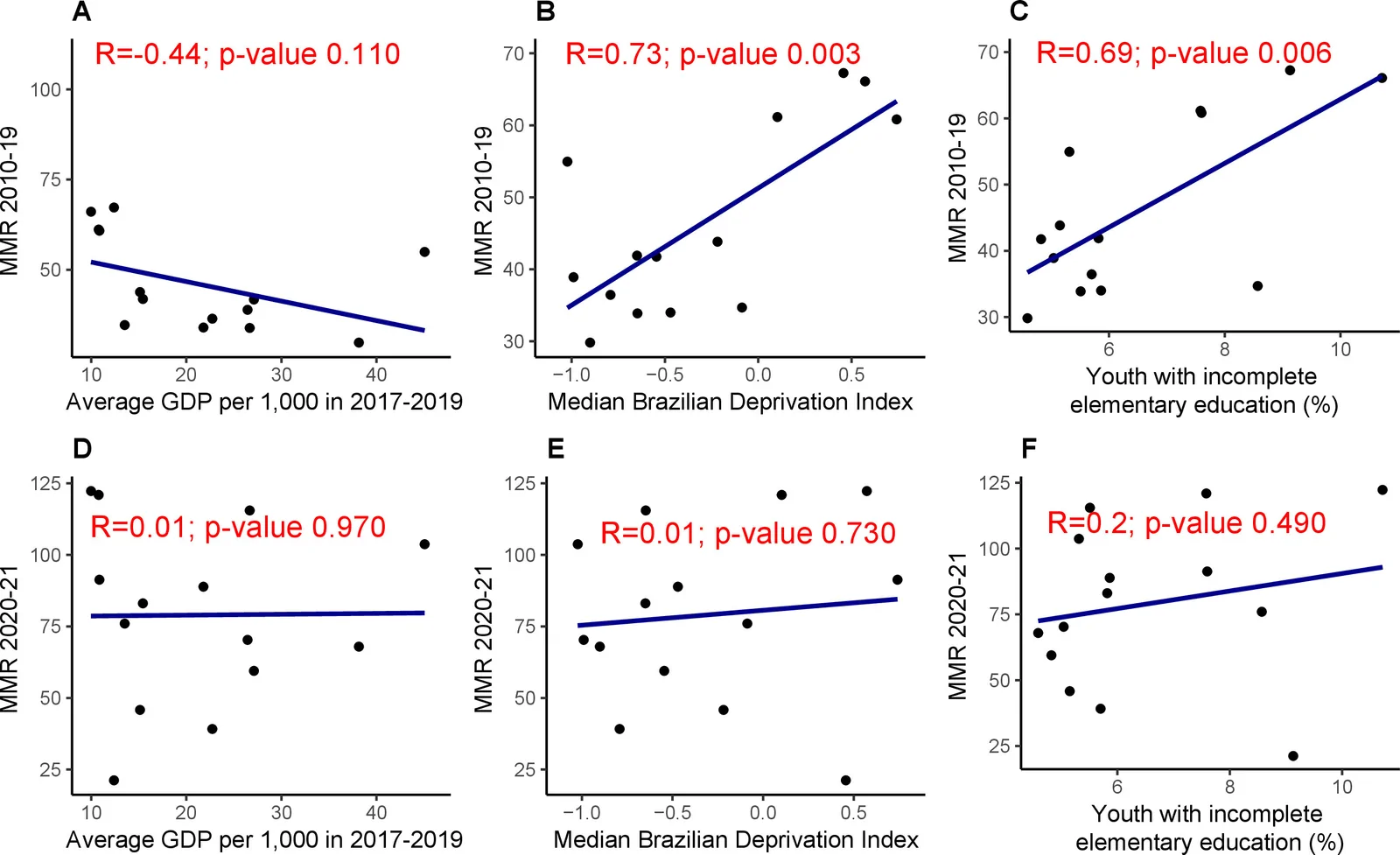
Estimated correlation using Pearson’s correlation coefficient (R) between the health macro-region Maternal Mortality Ratios (MMR) and average Gross Domestic Product (GDP) per capita before (A) and during the pandemic (D), median Brazilian Deprivation Index before (B) and during the pandemic (E), and percentage of young people with incomplete elementary education before (C) and during the COVID-19 pandemic (F). Minas Gerais, 2010-2021 (n=1,488)

There was a proportional increase in indirect causes of death during the pandemic (21.3% in 2010-2019 vs. 54.5% in 2020-2021), which is explained by deaths due to COVID-19 (40.2% of total deaths) (Table 2). There were no significant changes in the proportional share of other cause groups. The percentage of investigations into deaths of women of childbearing age did not show significant differences between the periods (94.0% versus 92.8%). However, the difference in the increase in maternal deaths after the investigation of deaths of women of childbearing age was significant (35.7% vs. 70.6%) (Supplementary Table 2).

**Table 2:** Distribution of maternal deaths during pregnancy, childbirth and the puerperium by cause type and cause group as per the 10th revision of the International Classification of Diseases (ICD-MM) before and during the COVID-19 pandemic. Minas Gerais, 2010-2021 (n=1,488)

	2010-2019	2020-2021
	n (%)	n (%)
**Type of cause **		
Direct	866 (76.0)	149 (42.8)
Indirect	243 (21.3)	189 (54.3)
Undetermined	31 (2.7)	10 (2.9)
**Cause group of deaths during pregnancy, childbirth and the puerperium **(ICD-MM)		
Direct		
Group 1 Pregnancy with abortive outcome	87 (7.6)	11 (3.2)
Group 2 Hypertensive causes	232 (20.4)	35 (10.1)
Group 3 Obstetric hemorrhage	208 (18.3)	52 (14.9)
Group 4 Pregnancy-related infection	100 (8.8)	5 (1.4)
Group 5 Other obstetric complications	232 (20.4)	43 (12.4)
Group 6 Unanticipated complications (anesthesia-related)	7 (0.6)	3 (0.9)
Indirect		
Group 7 Non-obstetric complications	243 (21.3)	189 (54.3)
COVID-10 maternal deaths	0	140 (40.2^a^)
Group 8 Undetermined	31 (2.7)	10 (2.9)

^a^In relation to all causes of death in the period

## Discussion

The results found in this study indicate a significant increase in the MMR in Minas Gerais during the pandemic, with a heterogeneous impact among population groups and regions of the state. The increase was more pronounced among White women, young women, women with lower levels of education, women who had fewer prenatal consultations and women living in the Northwest and South Central macro-regions. It was also observed that socioeconomic inequalities, expressed by the Brazilian Deprivation Index and low levels of education, were already associated with higher MMRs in the period prior to the pandemic. There was a significant increase in maternal mortality after investigating deaths of women of childbearing age, especially during the pandemic, due to COVID-19.

This study has limitations, such as the lack of use of techniques designed to address the instability of unadjusted mortality rates in regions with a small number of deaths and to smooth out random fluctuations. Furthermore, the small number of deaths, due to the short observation period, did not allow for adjustments for confounders, which could explain some of the effects analyzed. Another limitation is that inherent to the quality of data taken from information systems. Although the Mortality Information System has good coverage, possible incompleteness in the filling in of sociodemographic variables should be considered. Also to be considered are changes in how the race/skin color variable was collected over the period analyzed, as it became self-reported, as well as changes in prenatal care, which may have affected temporal comparability.

The strain on services during the pandemic may have compromised completion of Death Certificates, potentially underestimating direct obstetric causes in women with COVID-19, where this infection may have been recorded as the underlying cause, to the detriment of other relevant obstetric conditions.

As this is an ecological study, the associations observed cannot be extrapolated to the individual level, as they are subject to ecological fallacy. However, this study had the strength of the good reliability of the data on maternal deaths held on the Mortality Information System in Minas Gerais. More than 93% of deaths in women of childbearing age during the study period were investigated, exceeding values ​​found nationally (below 90%) [7]. Furthermore, analysis of the original underlying cause versus the final underlying cause of death and distribution by cause groups revealed considerable underreporting of maternal deaths and showed that the increase was caused by COVID-19 itself.

The significant growth in the MMR in Minas Gerais was driven by the increase in indirect maternal deaths caused by COVID-19, which accounted for 40% of deaths and were the leading cause of maternal death in 2020-2021. Pregnancy causes physiological changes in the respiratory, cardiovascular, immune and coagulation systems, increasing the susceptibility of pregnant women to viral pneumonias [11]. Pregnant and postpartum women diagnosed with COVID-19 had more severe outcomes, such as a higher risk of admission to an intensive care unit (OR 5.84; 95%CI 5.46; 6.25), intubation and mechanical ventilation (OR 14.33; 95%CI 12.50; 16.42), than those without COVID-19 in the United States in 2020 and 2021 [20]. In Brazil, in 2020, the odds of hospitalization (OR 4.37; 95%CI 3.39; 5.37), ICU admission (OR 1.73; 95%CI 1.50; 1.98) and use of invasive ventilatory support (OR 1.64; 95%CI 1.42; 1.86) were higher in pregnant/postpartum women compared to non-pregnant/postpartum women of childbearing age who were infected [21]. Research conducted with 2,130 pregnant women in 18 countries revealed that risk of maternal death was substantially higher in the group with COVID-19 compared to the group of women without COVID-19 in 2020 [5]. Compared to 2020, pregnant and postpartum women had higher odds of death due to COVID-19 in Brazil in 2021 than non-pregnant/postpartum women and men [22].

In this study, women with lower education levels had the greatest increase and the highest MMR in both periods, with an MMR 2.8 times that of those with 8 or more years of schooling during the pandemic. Lower educational levels are generally associated with lower-paying occupations and informal and precarious work. Professions predominantly performed by women, especially Black and poor women, such as domestic work and caregiving activities, were considered essential during the pandemic. As a result, they could not follow social distancing recommendations, thus becoming more exposed to COVID-19 [23]. Furthermore, the higher maternal mortality in women with lower education levels may also have resulted from difficulties in timely access to healthcare services, ranging from early diagnosis to access to intensive care beds in severe cases. The relationship between education and maternal mortality expresses the important role of the social determinants of health in maternal mortality [24].

In this analysis, the highest MMRs occurred among women aged 35 or older and among Black women throughout the period, which is consistent with current literature [24,25]. However, the largest increases occurred among young women and those of White race/skin color. One hypothesis for the smaller increase in MMRs in groups that historically had higher MMRs is competing risk, in which the occurrence of certain events reduces or alters the probability of occurrence of another outcome [26]. In groups with historically higher MMRs, preventable causes tend to be more frequent, and COVID-19 may have competed with these causes, attenuating the increase observed. Regarding race/skin color, racial disparities in maternal mortality may be related to a higher risk of discrepancies in access to health services by the Black population. This occurs both in the quality of care received and in health outcomes [12], reflecting the unequal conditions promoted by structural racism. In Brazil, in 2015, Black women were subjected to less implementation of good practices in labor and other care moments [27], suggesting racial discrimination in the context of childbirth care.

There was a greater increase in the MMR in women who had fewer prenatal visits. It should be considered that prioritizing pandemic control resulted in restrictions on access to various health services, such as those related to sexual and reproductive health. Furthermore, non-COVID-19 related services were suspended, health professionals were reassigned and became ill, and women themselves reduced their demand for these services due to fear of contamination [23]. Prenatal care is fundamental for prevention and early recognition of diseases and complications. Non-use or less frequent use of this care can represent increased risks to the health of pregnant women.

Larger increases occurred in regions of the state that had the lowest MMRs before the pandemic, while regions with historically high MMRs showed more modest increases, emphasizing the hypothesis of competing risks [26]. COVID-19 may have competed with preventable causes of maternal mortality in regions with high MMRs, where the highest frequency of these causes is expected. The largest increases in MMRs may be related to higher incidence of COVID-19 in the macro-regions, which caused overcrowding in intensive care units and a lack of beds and supplies, in addition to the displacement of health professionals. It is important to consider that the variation in the availability and quality of health care between regions of the state may contribute to the inequalities in MMRs identified in this study.

The case of the Jequitinhonha macro-region is noteworthy because it had the highest MMR in the state in the pre-pandemic period, while during the pandemic it presented the lowest MMR. Although this region had a higher proportion of investigated deaths of women of childbearing age during the pandemic than it had before it (79.3% in 2010-2019 and 89.7% in 2020-2021), the small number of deaths in 2020-2021 (n=2) may be responsible for part of the observed effect. There is also the possibility of underreporting of maternal deaths, which could have underestimated the MMR in Jequitinhonha during that period.

Socioeconomic inequalities between regions of the state may be related to disparities in maternal mortality. The macro-regions with higher MMRs, further north in the state, are the least socioeconomically favored. When correlating socioeconomic indicators and the MMR of macro-region of residence, between 2010 and 2019 we found a strong correlation of the median health macro-region Brazilian Deprivation Index. This indicates that higher levels of material deprivation are associated with higher MMRs. Low family income and lack of access to drinking water and sanitation, among other elements of living conditions, are associated with higher MMRs [28].

In this analysis, the higher percentage of young people who did not complete elementary education was moderately correlated with higher MMRs in the macro-regions in 2010-2019 (R 0.69; p-value 0.006), indicating that the lower the levels of education, the higher the MMR. During the pandemic, no statistically significant associations were found between MMRs and socioeconomic indicators. One hypothesis for this is that the small number of deaths did not provide sufficient power to verify association. For example, in Brazil between 2020 and 2021, higher maternal mortality due to COVID-19 was demonstrated in municipalities with greater socioeconomic inequality than in areas with better social and infrastructure conditions [13].

In Brazil, the percentage of investigations into deaths of women of childbearing age fell from 88% in 2019 to 84% in 2020 [7], while in Minas Gerais it remained stable (94% vs. 93%). The 71% increase in the number of maternal deaths held on the Mortality Information System, after the investigation of deaths in women of childbearing age, suggests a worsening in the completion of maternal death certificates during the pandemic, with a consequent increase in underreporting. The demands of the pandemic, such as work overload, may have affected the accuracy of the records of the cause of death by the certifying professionals [29]. In this sense, during health crises, it is pertinent to develop official guidelines on the correct completion of maternal death certificates, with emphasis on the field for women of childbearing age and wide dissemination to professionals who certify death.

This study concluded that the significant increase in the MMR in Minas Gerais during the pandemic is attributable to maternal deaths due to COVID-19. Groups with historically lower MMRs experienced greater growth in the MMR compared to groups in which the indicator was higher. The MMR is a sensitive indicator of health conditions and the quality of care provided to women, and its increase during the pandemic reflects structural weaknesses in the obstetric care network and the effects of health crises on maternal health.

The results obtained in this analysis point to the need to promptly recognize pregnant and postpartum women as a risk group during epidemics related to respiratory infections; they need to be prioritized in immunization strategies. Furthermore, it is necessary to place greater emphasis on preventive measures such as maintaining sexual and reproductive health services, improving the quality of prenatal care, ensuring timely access to intensive care, rapidly identifying cases and continuously monitoring cases and deaths in the obstetric population, in order to reduce the risk of increased preventable maternal deaths.

## Data Availability

The data are available from the Minas Gerais State Health Department upon formal request and provided that the established ethical requirements are met.
