# Supplementary material for: Maternal mortality in the context of COVID-19: an ecological study comparing maternal mortality ratios before and during the pandemic, Minas Gerais, Brazil, 2010-2021
Source: Epidemiol Serv Saude. 2026 May 8;35:e20251008. doi: 10.1590/S2237-96222026v35e20251008.en (PMC13686423; doi:10.1590/S2237-96222026v35e20251008.en)
Supplement: Translation

# Translation: Mortalidade materna no contexto da covid-19: estudo ecológico com comparação das razões de mortalidade materna antes e durante a pandemia, Minas Gerais, 2010-2021

**DOI:** 10.1590/S2237-96222026v35e20251008.pt

**Authors:** Lívia Maria Gomes Lopes, Juliana Bottoni de Souza, Deborah Carvalho Malta, Flávia Bulegon Pilecco

**Affiliations:** 1 Universidade Federal de Minas Gerais Programa de Pós-Graduação em Saúde Pública Belo Horizonte MG Brasil Universidade Federal de Minas Gerais, Programa de Pós-Graduação em Saúde Pública, Belo Horizonte, MG, Brasil; 2 Universidade Federal de Minas Gerais Escola de Enfermagem Belo Horizonte MG Brasil Universidade Federal de Minas Gerais, Escola de Enfermagem, Belo Horizonte, MG, Brasil; 3 Universidade Federal de Minas Gerais Departamento de Enfermagem Materno Infantil e Saúde Pública Belo Horizonte MG Brasil Universidade Federal de Minas Gerais, Departamento de Enfermagem Materno Infantil e Saúde Pública, Belo Horizonte, MG, Brasil; 4 Universidade Federal de Minas Gerais Departamento de Medicina Preventiva e Social Belo Horizonte MG Brasil Universidade Federal de Minas Gerais, Departamento de Medicina Preventiva e Social, Belo Horizonte, MG, Brasil

**Keywords:** Mortalidade Materna, COVID-19, Pandemias, Disparidades nos Níveis de Saúde, Estudos Ecológicos

## Abstract

**Objetivo:**

Investigar mudanças na mortalidade materna durante a pandemia de covid-19 segundo características sociodemográficas, regionais e de atenção obstétrica em Minas Gerais e a correlação da Razão de Mortalidade Materna (RMM) com indicadores socioeconômicos da macrorregião de residência.

**Métodos:**

Estudo comparou dados do Sistema de Informações sobre Mortalidade entre 2010-2019 e 2020-2021. Calcularam-se a RMM e a variação percentual segundo idade, escolaridade, raça/cor da pele, número de consultas/trimestre de início do pré-natal e macrorregião de saúde. Avaliou-se a correlação entre RMM e variáveis socioeconômicas da macrorregião (Produto Interno Bruto [PIB] per capita, Índice Brasileiro de Privação e Escolaridade).

**Resultados:**

Foram registrados 1.488 óbitos. A RMM aumentou 62,5% na pandemia, especialmente em mulheres entre 20 e 34 anos (64,5%), brancas (77,7%), com menor escolaridade (134,9%), menor número de consultas pré-natal (102,2%), com início do pré-natal no 1º trimestre (58,1%) e residentes da macrorregião Noroeste (241%). Contudo, as mais elevadas RMM foram em mulheres de 35 anos ou mais (125,1/100 mil nascidos vivos), pretas (90,5/100 mil nascidos vivos), de menor escolaridade (151,5/100 mil nascidos vivos), menor número de consultas de pré-natal (181,2/100 mil nascidos vivos), que iniciaram o pré-natal no 1° trimestre (61,8/100 mil nascidos vivos) e residentes da macrorregião Nordeste (122,3/100 mil nascidos vivos). Não encontramos correlação entre os indicadores socioeconômicos e RMM na pandemia.

**Conclusão:**

O aumento percentual da razão na pandemia foi maior nos grupos e regiões com RMM historicamente mais baixa. O reconhecimento oportuno de gestantes/puérperas como grupo de risco em crises sanitárias pode reduzir mortes maternas evitáveis.

**Aspectos éticos**

Esta pesquisa respeitou os princípios éticos, obtendo os seguintes dados de aprovação:

Comitê de ética em pesquisa: Universidade Federal de Minas Gerais

Número do parecer: 5.910.012

Data de aprovação: 24/2/2023

Certificado de apresentação de apreciação ética: 66320122.1.0000.5149

Registro do consentimento livre e esclarecido: Dispensado.

## Introdução

A mortalidade materna é um recorrente problema de saúde pública, sobretudo em países de baixa e média renda. Na América Latina, nove a cada dez mortes são evitáveis, se forem aplicadas medidas e recomendações comprovadamente eficazes, tais como assistência materna de qualidade (inclusive diante emergências obstétricas), acesso universal a métodos contraceptivos e enfrentamento às desigualdades no acesso à saúde [1].

A pandemia de covid-19 afetou desproporcionalmente gestantes e puérperas, incrementando a Razão de Mortalidade Materna (RMM), especialmente em países em desenvolvimento, incluindo o Brasil [2-5], onde a RMM passou de 57,9 em 2019 para 74 óbitos por 100 mil nascidos vivos em 2020 [6] e para 117,4 óbitos por 100 mil nascidos vivos em 2021 [7]. Esse aumento da RMM distancia o país da meta de 30 óbitos por 100 mil nascidos vivos até 2030, pactuada nos Objetivos do Desenvolvimento Sustentável pelo Brasil, junto à Organização das Nações Unidas [8].

Além das mortes maternas provocadas diretamente pela covid-19, considera-se que a crise econômica e as restrições de investimentos públicos em saúde intensificaram os problemas pré-existentes no sistema de saúde no Brasil [9], alavancando efeitos indiretos da pandemia. O acesso a diversos serviços de saúde foi impactado pela sobrecarga e pelo desvio de profissionais de saúde, de leitos e de recursos financeiros para o enfrentamento à doença [10]. Em relação à atenção obstétrica, houve agravamento de problemas crônicos, como recursos insuficientes, limitações e desigualdades no acesso à assistência pré-natal qualificada e aos cuidados intensivos [11]. Ademais, as omissões e a negligência do governo Federal na condução da pandemia, a disseminação de notícias falsas e de medicamentos comprovadamente ineficazes e a demora na aquisição de vacinas para o controle da epidemia [2] contribuíram para o agravamento da crise no país.

Foram identificados riscos diferenciais na mortalidade materna relacionados a fatores socioeconômicos [4,12] e demográficos [3,13] em contextos nacionais durante a pandemia. Até o momento, não foram localizados estudos que explorassem variações temporais na RMM relacionadas à pandemia de covid-19 em um estado brasileiro tão heterogêneo como Minas Gerais e que não tem alcançado reduções importantes na RMM nos últimos anos [6]. No entanto, Minas Gerais apresenta boa confiabilidade nos dados sobre mortes maternas, com percentuais de investigação de óbitos de mulheres em idade fértil acima de 95% até 2019 [7]. Esta investigação permite a recuperação de mortes maternas não declaradas originalmente, essencial para uma estimativa da RMM mais próxima da realidade.

O objetivo deste estudo foi investigar as mudanças na mortalidade materna durante a pandemia de covid-19 segundo características sociodemográficas, regionais e de atenção obstétrica em Minas Gerais e a correlação da RMM com indicadores socioeconômicos da macrorregião de residência. 

## Métodos

### Delineamento

Estudo ecológico com comparação temporal da mortalidade materna entre os períodos pré-pandêmico e pandêmico de covid-19 em Minas Gerais, entre 2010 e 2021.

### Contexto

Considera-se óbito materno aquele ocorrido durante ou até 42 dias após o término da gestação, cuja causa básica esteja codificada no Capítulo XV da 10ª revisão da Classificação Internacional de Doenças (CID-10) [14]. Foram considerados os códigos de O00 a O99, exceto o O96 e o O97, referentes à morte materna tardia e à morte por sequelas de causas obstétricas diretas. Incluíram-se ainda os códigos A34, B20-B24, F53, D39.2, E23.0, M83.0, desde que as mortes estivessem relacionadas com o estado gravídico-puerperal e tenham ocorrido em até 42 dias após o parto [14].

Foram comparados dois períodos: pré-pandêmico (2010-2019) e pandêmico (2020-2021). A adoção de uma série histórica de dez anos no período anterior à pandemia teve como objetivo garantir maior estabilidade estatística, decorrente de um número adequado de óbitos.

### Participantes 

Todos os óbitos maternos registrados no Sistema de Informações sobre Mortalidade, de mulheres residentes em Minas Gerais, ocorridos entre 2010 e 2021. Por se tratar de um estudo censitário de base populacional, não foi realizado cálculo amostral. De 2010 a 2021, foram registrados 1.488 óbitos maternos.

### Variáveis

Calculou-se a RMM (número de óbitos maternos/número de nascidos vivos no mesmo período x 100 mil) tanto para o período anterior à pandemia de covid-19 (2010-2019), quanto para o período pandêmico (2020-2021) e a variação percentual dessa RMM: [(RMM final - RMM inicial) / RMM inicial] x 100. A RMM e a variação percentual foram calculadas para as seguintes variáveis:

- idade (em anos: ≤19; 20-34; ≥ 35);
- escolaridade (em anos de estudo: 0-7; ≥ 8);
- raça/cor da pele (branca; preta; parda);
- número de consultas de pré-natal (0-6; ≥7);
- trimestre de realização da primeira consulta pré-natal (1º trimestre; 2º-3º trimestre); e
- macrorregião de saúde de acordo com o Plano Diretor de Regionalização de Minas Gerais (Centro; Centro Sul; Jequitinhonha; Leste; Leste do Sul; Nordeste; Noroeste; Norte; Oeste; Sudeste; Sul; Triângulo do Norte; Triângulo do Sul; Vale do Aço) [15].

Na variável raça/cor da pele, as categorias amarela (n=3) e indígena (n=6) não foram apresentadas na tabela de resultados das variáveis sociodemográficas devido ao pequeno número de observações no período. Na variável trimestre de início do pré-natal, não foi considerado o ano de 2010 por inconsistências do campo no Sistema de Informações sobre Nascidos Vivos.

### Fonte de dados e mensuração

Os dados do Sistema de Informações sobre Mortalidade, incluindo as variáveis do módulo de investigação do óbito materno, foram obtidos mediante solicitação formal à Secretaria de Estado de Saúde de Minas Gerais. Os dados foram disponibilizados em março de 2023. O número de nascidos vivos, utilizado para o cálculo da RMM, foi acessado no Sistema de Informações sobre Nascidos Vivos, disponível no Sistema de Informações de Saúde do Departamento de Informática do Sistema Único de Saúde [7].

### Análise estatística 

Foram construídos gráficos do tipo mapas de calor para apresentar as RMM e os percentuais de mudança das RMM por macrorregiões de saúde, em que a cor varia em tonalidades de vermelho (Figura 1). À medida que a tonalidade do vermelho se intensifica, mais elevadas as RMM ou maiores incrementos percentuais. Por outro lado, tons menos intensos correspondem a menores RMM e a maior redução ou menores incrementos percentuais. 

**Figura 1:** Razões de mortalidade materna nas macrorregiões de saúde antes e durante a pandemia de covid-19 e os percentuais de mudança (PC) entre os períodos. Minas Gerais, 2010-2021 (n=1.488)

Para descrever as disparidades socioeconômicas entre as regiões, utilizamos o Produto Interno Bruto (PIB) per capita [16], o Índice Brasileiro de Privação [17] e o percentual de jovens (18-24 anos) que não completaram o ensino fundamental (baixa escolaridade). Os valores do PIB per capita médio (2017-2019) foram obtidos da base de dados do Instituto Brasileiro de Geografia e Estatística (IBGE) [18]; o Índice Brasileiro de Privação mediano, do Centro de Integração de Dados e Conhecimentos para Saúde [17]; e o percentual de escolarização, do Sistema de Informações de Saúde do Departamento de Informática do Sistema Único de Saúde (dados referentes ao censo 2010 do IBGE) [7]. Foi realizada uma análise gráfica da correlação entre esses indicadores e a RMM por macrorregião de saúde nos períodos analisados, calculando-se, adicionalmente, o coeficiente de correlação de Pearson (R) [19], adotando-se nível de significância de 5%. As análises foram conduzidas no software Stata, versão 14 e no software R com auxílio do pacote Rgeoda.

Por fim, descreveram-se o percentual de investigação e a proporção de óbitos de mulheres em idade fértil recategorizados como óbitos maternos pós-investigação, a partir da comparação da causa básica original e da causa básica final. Foram descritas a distribuição proporcional de óbitos segundo o tipo de causa (direta, indireta e indeterminada) e por grupos da CID-10 para óbitos na gravidez, parto e puerpério (CID-MM) [14] (Tabela suplementar 1). A CID-MM define, em um subgrupo, causas de morte que não deveriam ser utilizadas como causa básica. Neste estudo, elas foram realocadas para o grupo 5 – outras complicações obstétricas. Os óbitos maternos por covid-19 são identificados pelas seguintes causas básicas: O98.5 + U07.1; O98.5 + U07.2; O98.5 + U09.9; O99.8 + U10.9; O98.5 + U92.1 [6]. 

## Resultados

Foram avaliadas 1.488 declarações de óbito entre 2010 a 2021. Em Minas Gerais houve importante crescimento da RMM com a pandemia (62,5%), passando de 43,8 (2010-2019) para 71,1 óbitos por 100 mil nascidos vivos (2020-2021). Os maiores aumentos ocorreram entre mulheres de 20 a 34 anos (64,5%), com menor escolaridade (até 7 anos de estudo) (134,9%), raça/cor da pele branca (77,7%) e parda (65,7%), com menor número de consultas de pré-natal (0-6 consultas) (102,2%) e com início do pré-natal no 1º trimestre (58,1%). As maiores RMM durante a pandemia, no entanto, foram em mulheres de 35 anos ou mais (125,1 por 100 mil nascidos vivos), com menos de 8 anos de estudo (151,5 por 100 mil nascidos vivos), de raça/cor da pele preta (90,5 por 100 mil nascidos vivos), que realizaram 0 a 6 consultas de pré-natal (181,2 por 100 mil nascidos vivos) e que iniciaram o pré-natal no primeiro trimestre (61,8 por 100 mil nascidos vivos) (Tabela 1). 

**Tabela 1:** Razão de Mortalidade Materna (RMM) específica (por 100 mil nascidos vivos) segundo variáveis selecionadas antes e durante a pandemia de covid-19 e variação percentual entre os períodos. Minas Gerais, 2010-2021 (n=1.488)

Variáveis sociodemográficas e assistenciais	2010-2019 RMM (n)	2020-2021 RMM (n)	Variação entre períodos %
Total	43,8 (1,140)	71,1 (348)	62,5
**Faixa etária **(anos)			
≤19	25,9 (102)	34,6 (19)	33,6
20-34	38 (695)	62,5 (214)	64,5
≥35	89,7 (343)	125,1 (115)	39,5
**Escolaridade **(anos)^a^			
0-7	64,5 (342)	151,5 (77)	134,9
≥8	27,9 (563)	53,6 (234)	92,1
**Raça/cor da pele^b^**			
Brancas	40,4 (365)	71,8 (113)	77,7
Pretas	88,5 (162)	90,5 (43)	2,3
Pardas	41,4 (553)	68,6 (185)	65,7
**Consultas pré**-natal^c^			
0-6	89,6 (596)	181,2 (177)	102,2
≥7	24,4 (470)	40,4 (158)	65,6
**Trimestre de início do pré-natal**^d^			
1º	39,1 (689)	61,8 (254)	58,1
2º-3º	43,1 (146)	56,9 (34)	32,0

^a^ O campo em branco/ignorado correspondeu a 20,6% (2010-2019) e a 10,6% (2020-2021); ^b^ O campo em branco/ignorado correspondeu a 4,7% (2010-2019) e a 1,2% (2020-2021); ^c^ O campo em branco/ignorado correspondeu a 6,5% (2010-2019) e a 3,7% (2020-2021); ^d^ O campo em branco/ignorado correspondeu a 18,1% (2010-2019) e a 17,2% (2020-2021).

Observa-se o crescimento da RMM no período pandêmico na maioria das macrorregiões. A exceção é Jequitinhonha, a única que apresentou redução. No período pandêmico, os maiores crescimentos foram nas macrorregiões Noroeste (241,0%) e Centro Sul (161,4%) e os menores nas macrorregiões Vale do Aço (4,6%) e Sul (7,5%). As RMM mais elevadas foram nas macrorregiões Nordeste (122,3 por 100 mil nascidos vivos) e Leste (120,9 por 100 mil nascidos vivos) (Figura 1). 

A análise da correlação mostrou que, no período de 2010-2019, a RMM aumentou com o crescimento do Índice Brasileiro de Privação mediano da macrorregião de saúde (R 0,73; p-valor 0,003) e com o maior percentual de jovens que não completaram o ensino fundamental (R 0,69; p-valor 0,006). Não houve correlação estatisticamente significativa com o PIB per capita (R -0,44; p-valor 0,110). No período pandêmico, não houve correlações estatisticamente significativas entre RMM e os indicadores socioeconômicos avaliados (Figura 2). Mesmo retirando Jequitinhonha da análise, em razão de ter apresentado resultado muito divergente do esperado no período da pandemia, a ausência de correlações estatisticamente significativas permaneceu nesse período.

**Figura 2:** Correlação estimada pelo coeficiente de correlação de Pearson (R) entre Razões de Mortalidade Materna (RMM) da macrorregião de saúde e Produto Interno Bruto (PIB) per capita médio antes (A) e durante a pandemia (D), Índice Brasileiro de Privação mediano antes (B) e durante a pandemia (E) e percentual de jovens com o ensino fundamental incompleto antes (C) e durante a pandemia de covid-19 (F). Minas Gerais, 2010-2021 (n=1.488)

Houve aumento proporcional das causas indiretas de óbito na pandemia (21,3% em 2010-2019 vs. 54,5% em 2020-2021), o que se explica pelos óbitos por covid-19 (40,2% do total de óbitos) (Tabela 2). Não houve mudanças importantes na participação proporcional dos demais grupos de causas. O percentual de investigação de óbitos de mulheres em idade fértil não apresentou importantes diferenças entre os períodos (94,0% versus 92,8%). Já a diferença no incremento dos óbitos maternos após a investigação de óbitos de mulheres em idade fértil foi significativa (35,7% vs. 70,6%) (Tabela suplementar 2). 

**Tabela 2:** Distribuição de óbitos maternos segundo tipo de causa e grupos da 10ª revisão da Classificação Internacional de Doenças para óbitos na gravidez, parto e puerpério (CID-MM) antes e durante a pandemia de covid-19. Minas Gerais, 2010-2021 (n=1.488)

Variáveis	2010-2019	2020-2021
	n (%)	n (%)
**Tipo de causa**		
Diretas	866 (76,0)	149 (42,8)
Indiretas	243 (21,3)	189 (54,3)
Indeterminadas	31 (2,7)	10 (2,9)
**Grupo de causa de doenças para óbitos na gravidez, parto e puerpério **(CID-MM)		
Diretas		
Grupo 1 Gravidez que termina em aborto	87 (7,6)	11 (3,2)
Grupo 2 Causas hipertensivas	232 (20,4)	35 (10,1)
Grupo 3 Hemorragias	208 (18,3)	52 (14,9)
Grupo 4 Infecções relacionadas à gravidez	100 (8,8)	5 (1,4)
Grupo 5 Outras complicações obstétricas	232 (20,4)	43 (12,4)
Grupo 6 Complicações não antecipadas (anestesia)	7 (0,6)	3 (0,9)
Indiretas		
Grupo 7 Complicações não obstétricas	243 (21,3)	189 (54,3)
Óbitos maternos por covid-19	0	140 (40,2^a^)
Grupo 8 Indeterminadas	31 (2,7)	10 (2,9)

^a^Em relação a todas as causas de óbito no período

## Discussão

Os resultados encontrados neste estudo indicam aumento expressivo da RMM em Minas Gerais durante a pandemia, com impacto heterogêneo entre grupos populacionais e regiões do estado. O aumento foi mais nítido entre as mulheres brancas, jovens, com menor escolaridade, que realizaram menos consultas pré-natal e residentes das macrorregiões Noroeste e Centro-Sul. Observou-se, ainda, que desigualdades socioeconômicas, expressas pelo Índice Brasileiro de Privação e pela baixa escolaridade, já se associavam a maiores RMM no período anterior à pandemia. Houve importante incremento da mortalidade materna após investigação de óbitos de mulheres em idade fértil, especialmente na pandemia, em função da covid-19. 

Este estudo apresenta limitações como a não utilização de técnicas destinadas a lidar com a instabilidade dos valores da razão bruta em regiões com pequeno número de óbitos e suavizar flutuações aleatórias. Além disso, o pequeno número de óbitos, em razão do curto período de observação, não permitiu ajustes para confundidores, que podem explicar parte dos efeitos analisados. Outra limitação é aquela intrínseca à qualidade dos dados de sistemas de informação. Apesar de o Sistema de Informações sobre Mortalidade apresentar boa cobertura, devem ser consideradas possíveis incompletudes no preenchimento de variáveis sociodemográficas. Consideram-se também as mudanças na coleta da variável raça/cor da pele ao longo do período analisado, que passou a ser autorreferida, além de mudanças na assistência pré-natal, o que pode ter afetado a comparabilidade temporal. 

A sobrecarga dos serviços durante a pandemia pode ter comprometido o preenchimento da Declaração de Óbito, com possível subestimação de causas obstétricas diretas em mulheres com covid-19, nas quais a infecção pode ter sido registrada como causa básica, em detrimento de outras condições obstétricas relevantes. 

Em se tratando de estudo ecológico, as associações observadas não podem ser extrapoladas para o nível individual, estando sujeitas à falácia ecológica. No entanto, este estudo teve como ponto forte a boa confiabilidade nos dados sobre mortes maternas no Sistema de Informações sobre Mortalidade em Minas Gerais. A investigação de óbitos em mulheres em idade fértil no período estudado foi de mais de 93%, superando valores encontrados nacionalmente (inferiores a 90%) [7]. Além disso, a análise da causa básica original versus causa básica final de óbito e da distribuição por grupos de causas evidenciou considerável subnotificação de óbitos maternos e mostrou que o aumento foi causado pela própria covid-19. 

O importante crescimento da RMM em Minas Gerais foi alavancado pelo crescimento de óbitos maternos indiretos, causados pela covid-19, que representaram 40% das mortes e foram a principal causa de morte materna em 2020-2021. A gestação provoca modificações fisiológicas nos sistemas respiratório, cardiovascular, imunológico e na coagulação, aumentando suscetibilidade das gestantes às pneumonias virais [11]. Gestantes e puérperas com diagnóstico de covid-19 apresentavam desfechos mais graves como o maior risco de admissão em unidade de terapia intensiva (OR 5,84; IC95% 5,46; 6,25), entubação e ventilação mecânica (OR 14,33; IC95% 12,50; 16,42) do que às sem covid-19 nos Estados Unidos em 2020 e 2021 [20]. No Brasil, em 2020, as chances de hospitalização (OR 4,37; IC95% 3,39; 5,37), admissão na UTI (OR 1,73; IC95% 1,50; 1,98) e uso de suporte ventilatório invasivo (OR 1,64; IC95% 1,42; 1,86), foram maiores nas grávidas/puérperas em comparação a mulheres em idade fértil não grávidas/puérperas infectadas [21]. Pesquisa realizada com 2.130 grávidas em 18 países revelou que o risco de morte materna foi substancialmente maior no grupo com covid-19 em relação ao grupo de mulheres sem covid-19 em 2020 [5]. Se comparado a 2020, as grávidas e puérperas tiveram mais chance de morte por covid-19 no Brasil em 2021 do que as mulheres não grávidas/puérperas e homens [22]. 

Neste estudo, mulheres com menor escolaridade tiveram o maior aumento e as maiores RMM em ambos os períodos, com RMM 2,8 vezes a das com 8 anos ou mais de escolaridade durante a pandemia. Níveis educacionais mais baixos em geral estão associados a ocupações de menor remuneração e ao trabalho informal e precário. As profissões exercidas majoritariamente por mulheres, especialmente negras e pobres, como o trabalho doméstico e as atividades de cuidado, foram consideradas essenciais no período pandêmico. Como resultado, elas não puderam seguir as recomendações de distanciamento social, ficando mais expostas à covid-19 [23]. Além disso, a maior mortalidade materna em mulheres de menor escolaridade também pode ter decorrido de dificuldades de acesso oportuno a serviços de atenção à saúde, desde o diagnóstico precoce ao acesso a leitos de terapia intensiva nos casos graves. A relação entre escolaridade e mortalidade materna, exprime o importante papel dos determinantes sociais da saúde na mortalidade materna [24]. 

Nesta análise, as maiores RMM ocorreram entre mulheres de 35 anos ou mais e em mulheres pretas em todo o período, o que é condizente com a literatura corrente [24,25]. No entanto, os maiores aumentos ocorreram entre mulheres jovens e da raça/cor da pele branca. Uma hipótese para o menor aumento da RMM em grupos que apresentavam historicamente RMM mais elevada é a competição de riscos, no qual a ocorrência de determinados eventos reduz ou altera a probabilidade de ocorrência de outro desfecho [26]. Nos grupos com RMM historicamente mais elevada, as causas evitáveis tendem a ser mais frequentes, e a covid-19 pode ter competido com essas causas, atenuando o aumento observado. Em relação à raça/cor da pele, as disparidades raciais na mortalidade materna podem relacionar-se ao maior risco de discrepâncias no acesso aos serviços de saúde para a população negra. Isso se dá tanto na qualidade dos cuidados recebidos como nos resultados de saúde [12], como reflexo das condições desiguais promovidas pelo racismo estrutural. No Brasil, em 2015, mulheres pretas foram submetidas a menor realização de boas práticas na atenção ao trabalho de parto e outros momentos assistenciais [27], sugerindo discriminação racial no contexto de atenção ao parto.

Houve maior crescimento da RMM em mulheres que haviam realizado menor número de consultas pré-natal. Deve-se considerar que a priorização do controle da pandemia resultou em restrições de acesso a diversos serviços de saúde como, por exemplo, os relacionados à saúde sexual e reprodutiva. Ademais, suspenderam-se atendimentos não relacionados à covid-19, houve remanejamento e adoecimento de profissionais de saúde, além do fato de a procura desses serviços pelas próprias mulheres ter reduzido pelo medo da contaminação [23]. A assistência pré-natal é fundamental na prevenção e no reconhecimento precoce de doenças e complicações. A não utilização ou a menor frequência na utilização dessa assistência pode representar aumento de riscos para a saúde da gestante. 

Ocorreram maiores aumentos em regiões do estado que tinham as mais baixas RMM antes da pandemia, enquanto regiões com RMM historicamente elevadas apresentaram incrementos mais modestos, o que reforça a hipótese de riscos competitivos [26]. A covid-19 pode ter competido com as causas evitáveis de mortalidade materna em regiões com elevadas RMM, onde se espera a maior frequência dessas causas. Os maiores aumentos da RMM podem estar relacionados a maiores incidências de covid-19 nas macrorregiões, o que provoca superlotação em unidades de terapia intensiva e falta de leitos e de insumos, além do deslocamento de profissionais. É importante considerar que a variação na disponibilidade e qualidade da assistência à saúde entre regiões do estado pode contribuir para as desigualdades na RMM identificadas neste estudo. 

O caso da macrorregião Jequitinhonha chama a atenção, pois, tinha a maior RMM do estado no período pré-pandêmico e, durante a pandemia, apresentou a menor RMM. Embora essa região tenha tido, na pandemia, uma proporção de investigação de óbito de mulheres em idade fértil superior ao que vinha apresentando antes (79,3% em 2010-2019 e 89,7% em 2020-2021), o pequeno número de óbitos em 2020-2021 (n=2) pode ser responsável por parte do efeito observado. Há ainda a possibilidade de subnotificação de óbitos maternos, o que poderia ter subestimado a RMM de Jequitinhonha no período.

As desigualdades socioeconômicas entre as regiões do estado podem estar relacionadas com disparidades na mortalidade materna. As macrorregiões com maiores RMM, mais ao norte do estado, são as menos favorecidas socioeconomicamente. Ao correlacionar indicadores socioeconômicos e a RMM da macrorregião de residência, entre 2010 e 2019 encontramos correlação forte do Índice Brasileiro de Privação mediano da macrorregião. Isso indica que maiores níveis de privação material se associam a RMM mais elevadas. O baixo rendimento familiar e a falta de acesso a água potável e saneamento, entre outros elementos das condições de vida, associam-se à maior RMM [28]. 

Nesta análise, o maior percentual de jovens que não completaram o ensino fundamental foi moderadamente correlacionado à maior RMM nas macrorregiões em 2010-2019 (R 0,69; p-valor 0,006), sinalizando que quanto menor os níveis de escolaridade, maior a RMM. Durante a pandemia, não foram encontradas associações estatisticamente significativas da RMM com os indicadores socioeconômicos. Uma hipótese é que o pequeno número de óbitos não conferiu poder suficiente para verificar associação. Por exemplo, no Brasil entre 2020 e 2021, demonstrou-se maior mortalidade materna por covid-19 em municípios com maior desigualdade socioeconômica do que em áreas com melhores condições sociais e de infraestrutura [13]. 

No Brasil, o percentual de investigação de óbitos de mulheres em idade fértil caiu de 88% em 2019 para 84% em 2020 [7], enquanto em Minas Gerais manteve-se estável (94% vs. 93%). O incremento de 71% no número de óbitos maternos no Sistema de Informações sobre Mortalidade, após a investigação de óbitos em mulheres em idade fértil, sugere a piora no preenchimento das declarações de óbitos maternos durante a pandemia, com consequente aumento de subnotificações. As demandas da pandemia, como a sobrecarga de trabalho, podem ter afetado a precisão dos registros da causa de morte pelos profissionais atestantes [29]. Nesse sentido, durante crises sanitárias, é pertinente elaborar orientações oficiais sobre o correto preenchimento das declarações de óbitos maternos, com ênfase no campo para mulheres em idade fértil e ampla divulgação aos profissionais que atestam o óbito.

Este estudo concluiu que o aumento significativo da RMM em Minas Gerais durante a pandemia é atribuível aos óbitos maternos por covid-19. Grupos com RMM historicamente mais baixa experimentaram maior crescimento da RMM em comparação aos grupos nos quais o indicador era mais elevado. A RMM é um indicador sensível das condições de saúde e da qualidade da atenção prestada às mulheres, cuja elevação durante a pandemia reflete fragilidades estruturais da rede de atenção obstétrica e os efeitos das crises sanitárias sobre a saúde materna. 

Os resultados alcançados nesta análise apontam para a necessidade de reconhecer prontamente as gestantes e puérperas como grupo de risco durante epidemias relacionadas a infecções respiratórias; é preciso priorizá-las em estratégias de imunização. Ademais, faz-se necessário dar mais ênfase em medidas preventivas tais como manutenção dos serviços de saúde sexual e reprodutiva, aumento da qualidade dos cuidados pré-natais, acesso em tempo hábil aos cuidados intensivos, identificação rápida dos casos e monitoramento contínuo dos casos e óbitos na população obstétrica, de modo a reduzir o risco do aumento das mortes maternas evitáveis. 

## Data Availability

Os dados são disponibilizados pela Secretaria de Estado de Saúde de Minas Gerais, mediante solicitação formal e desde que atendidos os requisitos éticos estabelecidos.
